# Quantitative isoform-profiling of highly diversified recognition molecules

**DOI:** 10.7554/eLife.07794

**Published:** 2015-05-18

**Authors:** Dietmar Schreiner, Jovan Simicevic, Erik Ahrné, Alexander Schmidt, Peter Scheiffele

**Affiliations:** 1 Biozentrum, University of Basel, Basel, Switzerland; Stanford School of Medicine, United States

**Keywords:** alternative splicing, SRM, MRM, synapse, neuroligin, recognition, mouse

## Abstract

Complex biological systems rely on cell surface cues that govern cellular self-recognition and selective interactions with appropriate partners. Molecular diversification of cell surface recognition molecules through DNA recombination and complex alternative splicing has emerged as an important principle for encoding such interactions. However, the lack of tools to specifically detect and quantify receptor protein isoforms is a major impediment to functional studies. We here developed a workflow for targeted mass spectrometry by selected reaction monitoring that permits quantitative assessment of highly diversified protein families. We apply this workflow to dissecting the molecular diversity of the neuronal neurexin receptors and uncover an alternative splicing-dependent recognition code for synaptic ligands.

**DOI:** http://dx.doi.org/10.7554/eLife.07794.001

## eLife digest

To create a protein, a gene is first copied to form an RNA molecule that contains regions known as introns and exons. Splicing removes the introns and joins the exons together to form a molecule of ‘messenger RNA’, which is translated into a protein.

Over the course of evolution, many groups—or families—of proteins have expanded and diversified their roles. One way in which this can occur is through a process known as alternative splicing, in which different exons can be included or excluded to generate the final messenger RNA. In this way, a single gene can produce a number of different proteins. These closely related proteins are known as isoforms.

The brain contains billions of neurons that communicate with one another across connections known as synapses. A family of proteins called neurexins helps neurons to form these synapses. Humans have three neurexin genes, which undergo extensive alternative splicing to produce thousands of protein isoforms. However, it is not known whether all of these isoforms are produced in neurons, as existing experimental techniques were not sensitive enough to easily distinguish one isoform from another.

A technique known as ‘selected reaction monitoring’ (or SRM for short) has recently emerged as a promising way to identify proteins. This allows proteins containing specific sequences to be separated out for analysis, in contrast to existing techniques that test randomly selected protein samples, which will result in most isoforms being missed. Schreiner, Simicevic et al. have now developed SRM further and show that this technique can detect the identity and amount of the neurexin isoforms present at synapses, including those that are only produced in very small quantities. Using SRM, Schreiner, Simicevic et al. demonstrate that neurexin isoforms differ in how they interact with synaptic receptors. Thus, alternative splicing of neurexins underlies a ‘recognition code’ at neuronal synapses.

In the future, this newly developed SRM method could be used to investigate isoforms in other protein families and tissues, and so may prove valuable for understanding how a wide range of cellular recognition processes work.

**DOI:** http://dx.doi.org/10.7554/eLife.07794.002

## Introduction

The remarkable anatomical and functional complexity of nervous systems relies on molecular programs for cell intrinsic properties and selective cellular interactions. Major advances in transcriptomics have enabled the identification of gene regulatory programs and mRNA targets that underlie specification of neuronal cell types and their plasticity (Hobert, 2011; Ebert and Greenberg, 2013; Molyneaux et al., 2015). For example, specific transcriptional programs direct the neurotransmitter phenotypes of neuronal populations, the targeting of axonal projections, or the modification of synapse numbers in response to neuronal activity. In addition, transcript-based studies have uncovered gene families with substantial molecular complexity that may encode neuronal recognition events (Zipursky and Sanes, 2010; Schreiner et al., 2014a).

What remains a major challenge is the exploration of such molecular programs and their function at the protein level. mRNA and protein turnover rates as well as mRNA translation rates exhibit a significant dynamic range. Thus, transcript abundance cannot easily be extrapolated to provide quantitative assessments of the proteome or insights into the stoichiometry of protein complexes (Helbig et al., 2011; Schwanhausser et al., 2011; Vogel and Marcotte, 2012). Notably, such post-transcriptional forms of gene regulation are particularly prevalent in the central nervous system highlighting the need for quantitative approaches that enable targeted dissection of the neuronal proteome. Additionally, many neurons possess long-distance projections. The localization of presynaptic proteins in many cases differs from the anatomical place of their mRNA expression. Thus, the possibility to detect and quantify isoforms at the protein level provides an important advantage in order to understand the functional role of these proteins.

New developments in proteomics have driven major advances in understanding the cell biological mechanisms of neuronal development and connectivity (Bayes and Grant, 2009; Craft et al., 2013). These led to the delineation of the composition of purified synaptic vesicles, postsynaptic neurotransmitter receptor complexes, or models of presynaptic terminals (Takamori et al., 2006; Shanks et al., 2012; Schwenk et al., 2014; Wilhelm et al., 2014). Most of these studies employed semi-quantitative ‘shotgun’ mass spectrometry approaches (Liu et al., 2004; Venable et al., 2004; Neilson et al., 2011) that are based on the random sampling of peptide fragments detected in a sample. In this regard, the complexity of neuronal tissues and the molecular diversity of some neuronal receptor families pose significant limitations. First, many proteins are present in only a fraction of the cells or structures analyzed reducing the chance of detecting peptide levels required for adequate quantification. Second, over the past years alternative splicing has emerged as a key mechanism for the regulation of neuronal recognition (Aoto et al., 2013; Takahashi and Craig, 2013; He et al., 2014; Iijima et al., 2014; Lah et al., 2014). Alternative splicing programs can generate families of tens, hundreds, or even thousands of closely related protein isoforms frequently distinguished by only a single peptide. Thus, when applying random sampling (‘shotgun’) approaches it is challenging to obtain sufficient coverage for isoform detection and quantification. Additionally, the protein inference problem, i.e., presence of the same peptide sequence in multiple different proteins or protein isoforms limits applicability of ‘shotgun’ approaches for detection and quantification of protein families with high sequence homologies (Nesvizhskii and Aebersold, 2005).

One possibility to circumvent these problems is the application of targeted proteomic approaches, such as selected reaction monitoring (SRM; also referred to as MRM for ‘multiple reaction monitoring’). While originally developed for characterization of chemical compounds this method has recently emerged as promising technique for the quantitative analysis of protein species in biological samples (Phanstiel et al., 2008; Picotti and Aebersold, 2012; Carr et al., 2014). Instead of sampling a random portion of the proteome, SRM assays use optimized separation and detection parameters for a set of pre-selected peptides (termed proteotypic peptides or PTPs) that are specific to a protein or isoform of interest. PTPs are detected based on their chromatographic retention time and mass to charge ratio (rather than by sequencing as in shotgun proteomics). For each PTP, an isotopically labeled reference peptide is added to the sample which then is used as standard for quantification. This strategy greatly reduces experimental variability and significantly increases sensitivity in the detection and quantification of selected peptides (Bauer et al., 2014). To date, significant contributions in advancing our knowledge in biology have been made by applying SRM-based approaches, in particular to profile cellular pathways and metabolic states, thereby quantifying low abundance proteins within complex mixtures (Picotti et al., 2013; Simicevic et al., 2013; Kennedy et al., 2014). SRM assays have also been applied successfully to detect and quantify neuronal proteins (Zhang et al., 2012; Craft et al., 2013). However, the applicability for the dissection of highly diversified protein families in complex tissues remains to be explored.

In this study, we developed an array of SRM-based assays for the detection and quantification of highly diversified neuronal receptors. As a case study we focused on neurexins, a class of synaptic adhesion molecules which are widely expressed in the CNS and play an important role in synapse formation and function (Dean et al., 2003; Missler et al., 2003; Graf et al., 2004; Chih et al., 2006; Taniguchi et al., 2007; Uemura et al., 2010). In mammals, three neurexin genes (*Nrxn*1, 2, 3) are transcribed from two alternative promoters giving rise to long alpha-neurexin and short beta-neurexin transcripts (Reissner et al., 2013). Alternative splicing at six alternatively spliced segments (AS1-6) generates >1000 unique isoforms which can be detected in the adult brain at the transcript level (Baudouin and Scheiffele, 2010; Schreiner et al., 2014b). Importantly, insertion or skipping of alternative exons at two of the alternatively spliced segments (AS2 and AS4) was reported to regulate binding of neurexins to a number of different interaction partners (Ichtchenko et al., 1995; Sugita et al., 2001; Chih et al., 2006; Koehnke et al., 2010; Siddiqui et al., 2010; Uemura et al., 2010; Reissner et al., 2013). This raises the possibility that neurexin molecular diversity—and in particular its regulation by alternative splicing—may serve synaptic recognition events that control neuronal wiring and function.

The challenge with exploring isoform-specific functions is the lack of suitable tools to detect or quantify endogenous neurexin protein variants. Thus, even fundamental questions have remained unanswered: are neurexins abundant synaptic components? What is the relative contribution of the individual neurexin isoforms (NRX1, 2, 3 alpha and beta forms) to the total neurexin repertoire? Are proteins produced from all of the detected alternative transcripts, in particular transcripts encoding rare splice insertions? And does alternative splicing at all sites modify receptor–ligand interactions? Lastly, any studies on selective receptor–ligand interactions have relied on overexpressed or recombinant proteins as no assays are available to specifically probe or detect the endogenous protein isoforms.

In this study, we developed, validated, and applied a targeted proteomics workflow for the analysis of neurexin protein variants in the mouse brain using SRM-based assays. We provide a detailed protein expression map of neurexin alternative splice insertions, their absolute quantification, and uncover novel alternative splicing-dependent regulation of neurexin–ligand interactions. This workflow can be highly multiplexed and applied to virtually any protein family and tissue. Thus, it provides important new directions for the dissection of molecular diversity in cellular recognition and pathologies.

## Results

### Generation and characterization of peptide library and sample preparation

We established an array of targeted proteomic assays that allow for detection and quantitative determination of neurexin variants in complex samples. The approach includes: (1) In silico selection of proteotypic peptide candidates, (2) synthesis and experimental test of candidate-peptide performance in targeted mass spectrometry, and (3) identification of corresponding endogenous peptides by MS/MS sequencing analysis.

We used a customized sequence database containing all known neurexin variants derived from PacBio sequencing data (Schreiner et al., 2014b). A set of predicted tryptic peptide sequences was selected based on sequence uniqueness, size, and amino acid composition (Picotti et al., 2013; Bauer et al., 2014). Since neurexins are known to be glycosylated we examined peptides for the presence of consensus sequences for carbohydrate modifications and excluded peptides with potential post-translational modification from our assays. To evaluate the suitability of the selected peptides for MS detection, chemically synthesized peptides were analyzed on a triple-quadrupole LC-MS instrument. For each peptide, we determined retention times, fragmentation patterns and optimized collision energies in order to devise highly sensitive and specific SRM assays (Figure 1—figure supplement 1–7, see ‘Materials and methods’ for details).

**Figure 1.**
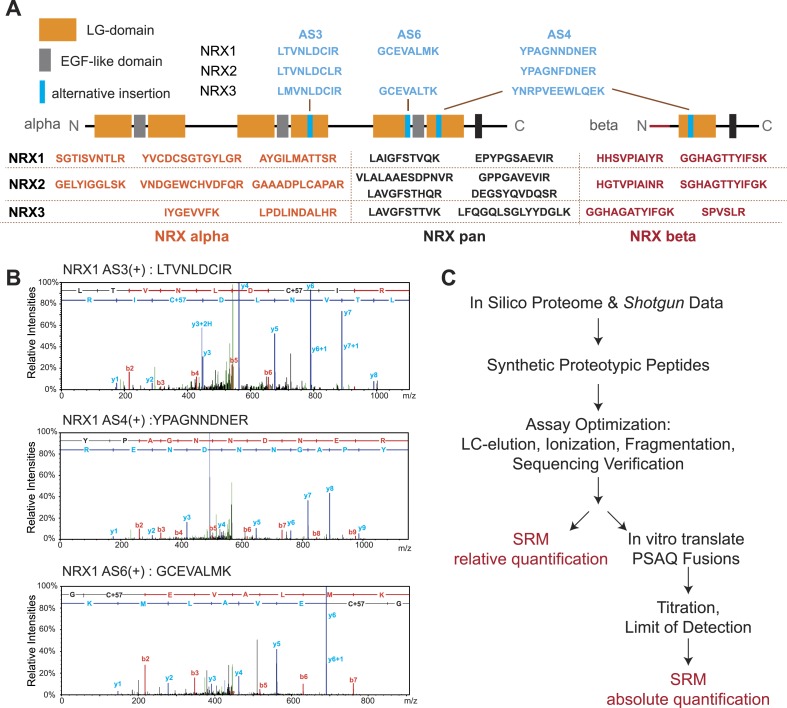
Establishment and validation of SRM-assays. (**A**) Proteotypic peptides for relative quantification of neurexin variants. The position of peptides within the overall domain structure of neurexin proteins is indicated (laminin-G and EGF-domains are marked, transmembrane domain (TMD) shown as box, sites modified by alternative splicing in blue). Validated pan-neurexin peptides shared amongst alpha and beta isoforms derived from each *Nrxn* gene are shown in black, neurexin-alpha specific peptides in orange, neurexin-beta specific peptides in red, and splice isoform-specific peptides in blue. (**B**) Example MS/MS spectra of three endogenous peptides used for detection and quantification of NRX1 splice variants containing insertions at alternatively spliced segments 3, 4, and 6. Letters above the peaks indicate the amino acid sequence of the corresponding peptides (blue = y-ions; red = b-ions). (**C**) Workflow for the quantitative SRM-based protein analysis. **DOI:** http://dx.doi.org/10.7554/eLife.07794.003

Peptide identification in SRM is based on the chromatographic properties, mass, and charge of proteotypic peptides but lacks the sequencing capabilities of shotgun approaches (Carr et al., 2014). Thus, we separately verified identities of peptides utilized for quantification by MS sequencing using LC-MS of endogenous neurexin proteins from mouse brain and immuno-precipitates obtained with a pan-NRX antibody (Figure 1B and Figure 1—figure supplement 1–7). In total, we generated and validated 30 SRM assays that enable detection and quantification of 17 specific NRX protein species with multiple PTPs per isoform wherever possible (Figure 1A): (a) NRX1, 2, and 3 total protein (pan-peptides), (b) alpha- and beta-protein variants derived from each *Nrxn* gene (alpha and beta-specific peptides), (c) alternative splice insertions at the alternatively spliced segments 3, 4, and 6. Note that several of the isoform-specific peptides differ in only a single amino acid residue between the NRX1, NRX2, and NRX3 isoforms making MS-based approaches the method of choice to specifically detect them. In addition, we established assays for 15 unrelated synaptic proteins to be used for comparison (the complete list of transitions shown in Supplementary file 1A).

**Figure 1—figure supplement 1.**
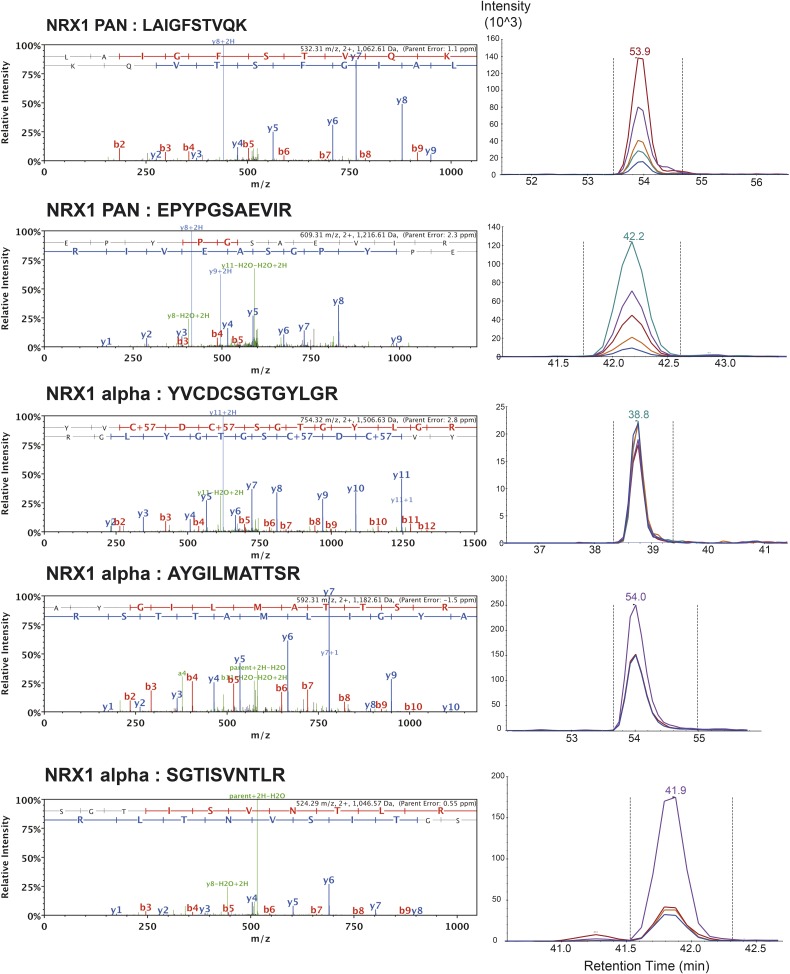
List of MS/MS spectra of proteotypic endogenous NRX1-pan and NRX1-alpha peptides, retention times and transition patterns of corresponding synthetic heavy peptides used for relative quantification in this study. **DOI:** http://dx.doi.org/10.7554/eLife.07794.004

**Figure 1—figure supplement 2.**
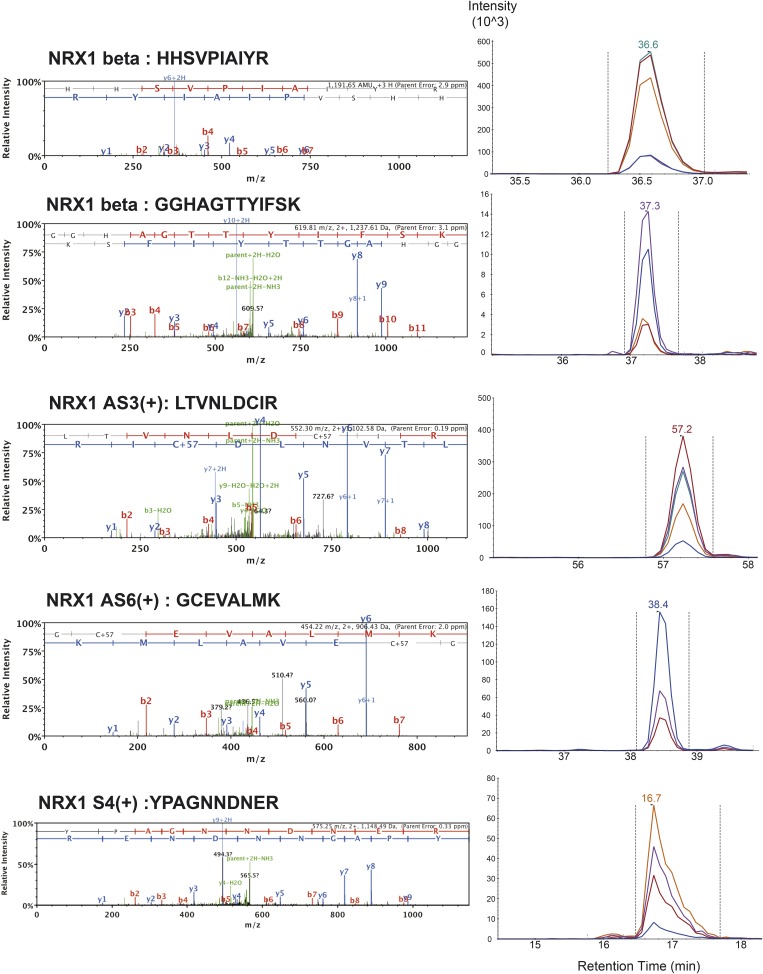
List of MS/MS spectra of proteotypic endogenous NRX1-beta, NRX1-AS3(+), NRX1-AS6(+) and NRX1-AS4(+) peptides, retention times and transition patterns of corresponding synthetic heavy peptides used for relative quantification in this study. **DOI:** http://dx.doi.org/10.7554/eLife.07794.005

**Figure 1—figure supplement 3.**
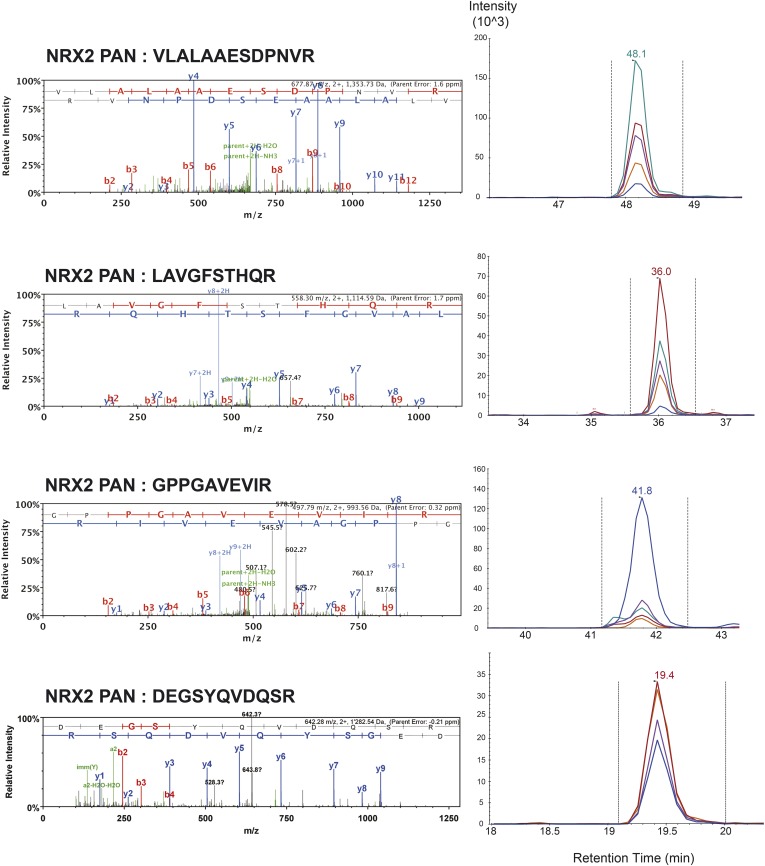
List of MS/MS spectra of proteotypic endogenous NRX2-pan peptides, retention times and transition patterns of corresponding synthetic heavy peptides used for relative quantification in this study. **DOI:** http://dx.doi.org/10.7554/eLife.07794.006

**Figure 1—figure supplement 4.**
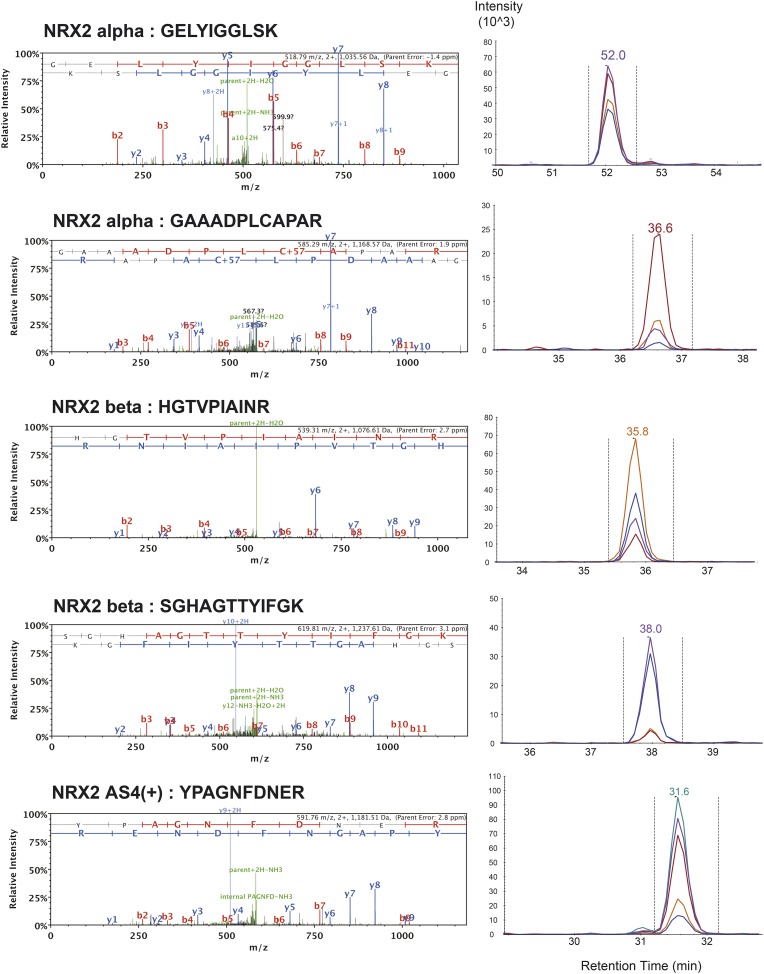
List of MS/MS spectra of proteotypic endogenous NRX2-alpha, NRX2-beta and NRX2-AS4(+) peptides, retention times and transition patterns of corresponding synthetic heavy peptides used for relative quantification in this study. Note: one NRX2-alpha specific peptide (VNDGEWCHVDFQR) could not be identified by shotgun MS/MS. However, read-outs obtained with this peptide were very close to values obtained with two other NRX2-alpha peptides. Thus, results obtained with all three peptides were used for relative quantification. **DOI:** http://dx.doi.org/10.7554/eLife.07794.007

**Figure 1—figure supplement 5.**
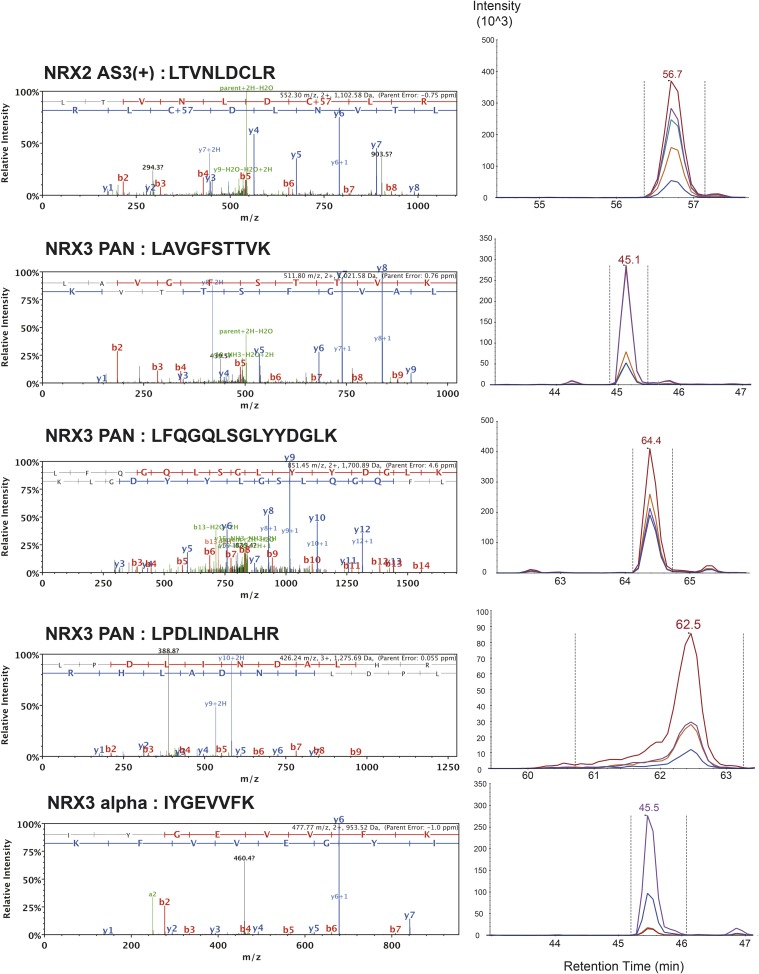
List of MS/MS spectra of proteotypic endogenous NRX2-AS3(+), NRX3-pan and NRX3-alpha peptides, retention times and transition patterns of corresponding synthetic heavy peptides used for relative quantification in this study. **DOI:** http://dx.doi.org/10.7554/eLife.07794.008

**Figure 1—figure supplement 6.**
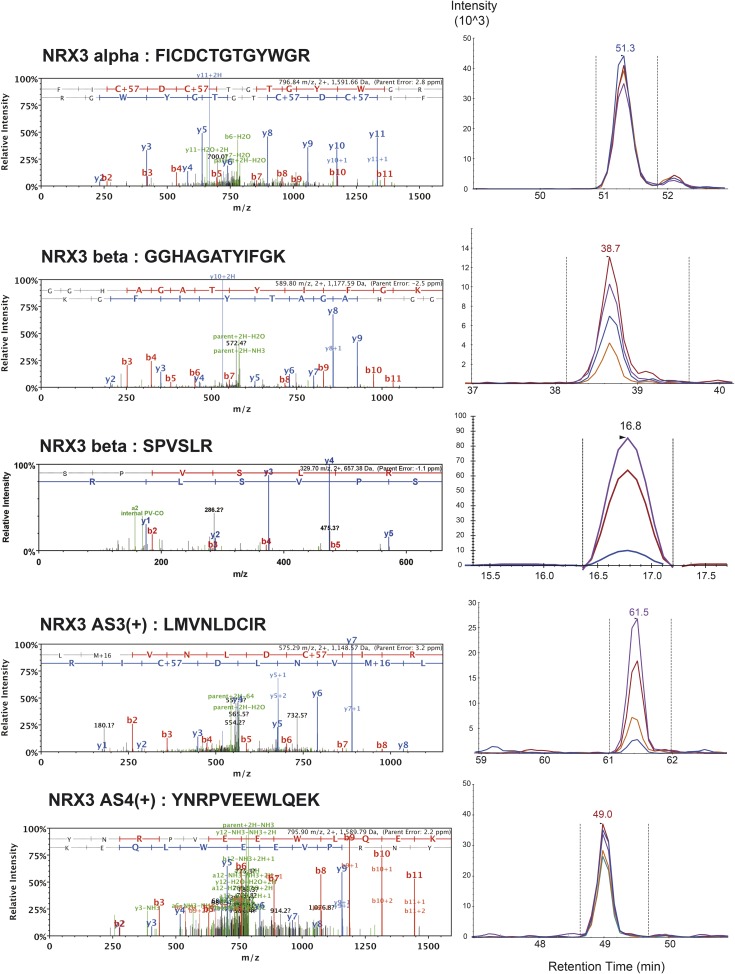
List of MS/MS spectra of proteotypic endogenous NRX3-alpha, NRX3-beta, NRX3-AS3(+) and NRX3-AS4(+) peptides, retention times and transition patterns of corresponding synthetic heavy peptides used for relative quantification in this study. **DOI:** http://dx.doi.org/10.7554/eLife.07794.009

**Figure 1—figure supplement 7.**
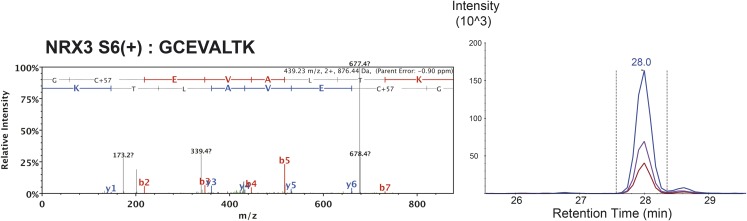
MS/MS spectrum of proteotypic endogenous NRX3-AS6(+) peptide, retention time and transition pattern of corresponding synthetic heavy peptide used for relative quantification in this study. **DOI:** http://dx.doi.org/10.7554/eLife.07794.010

To optimize detection of rare NRX isoforms, we sought a rapid enrichment strategy that would allow for high sample throughput and reliable SRM-based quantification. To this end, we isolated Triton X-100 resistant membrane fractions (TRM) from crude synaptosomes. We confirmed enrichment of major synaptic proteins by direct comparison to fractions obtained from conventional synaptosome preparations using shotgun mass spectrometry and quantitative SRM assays (Figure 2—figure supplement 1). The enrichment of neurexins was comparable with both protocols (Figure 2—figure supplement 2) and no significant bias was detected in the enrichment of alpha- and beta-isoforms (Figure 2—figure supplement 2). Most importantly, this simplified procedure is fast and yields from 3 mg of starting material ca. 1.5 µg of synaptic-enriched proteins sufficient for up to 100 SRM-assays. Thus, we used this simplified protocol for subsequent analyses of relative and absolute NRX protein levels across brain regions.

### Regional diversity of neurexin variants in adult mouse brain

**Figure 2.**
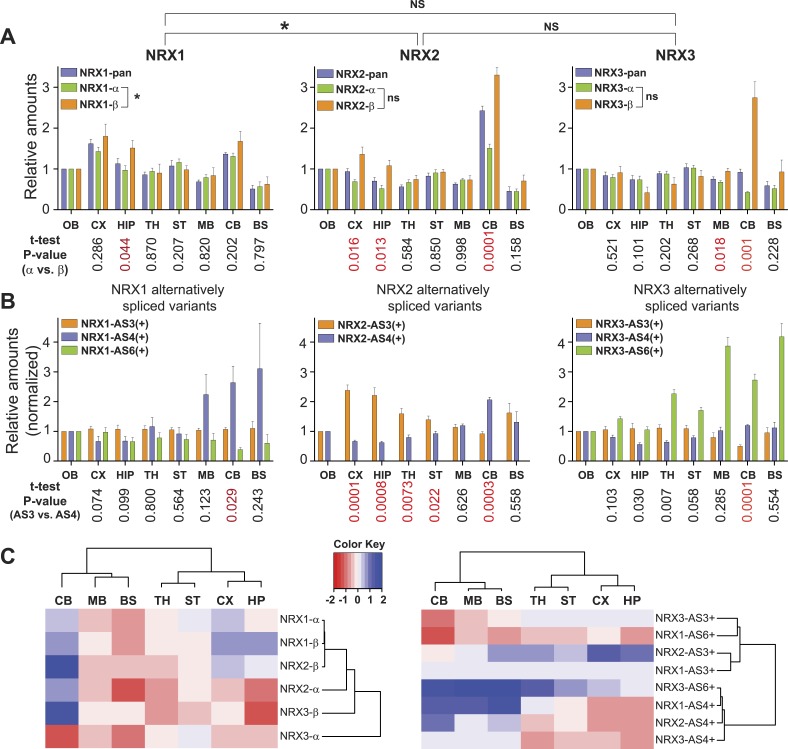
Quantitative comparison of relative neurexin variant levels across brain areas. (**A, B**) Relative amounts of total neurexin (NRX-pan), NRX-alpha, beta, and splice insertions across P30 mouse brain (OB = olfactory bulb, CX = cortex, HIP = hippocampus, TH = thalamus, ST = striatum, MB = mid-brain, CB = cerebellum, BS = brain stem). Values were normalized to OB. Correlation analysis between expression profiles of pan-neurexins, alpha and beta isoforms was performed using Spearman-correlation test. No significant correlation between expression profiles of alpha and beta isoforms of NRX2 and 3 (Spearman r = 0.6190, p-value = 0.1150 for NRX2 and Spearman r = −0.4048, p-value = 0.3268 for NRX3, respectively). Significant correlation (p-value = 0.0046 and Spearman r = 0.9048) for expression profiles of NRX1-alpha and beta isoforms. Numbers on the bottom of diagrams represent p-values of the t-test analysis of alpha- and beta-NRX in different brain areas (statistically significant values are marked in red). Relative amounts of neurexin AS3, AS4, AS6 splice variants across brain regions normalized to respective total NRX protein levels. As the AS3 and AS6 insertions are found exclusively in alpha variants, their measurements were normalized to NRX-alpha protein levels. Means ± SD from 4 biological replicates (n = 4, 2 male and 2 female mice, postnatal day 30) measured in 2 technical replicates. (**C**) Hierarchical clustering analysis of relative expression of NRX isoforms and splice variants across mouse brain. Hierarchical clustering of ‘protein log2 abundance ratios’ was performed using Ward's algorithm and the correlation distance metric. Subsequently, a heat map was created using the gplots R package. **DOI:** http://dx.doi.org/10.7554/eLife.07794.011

To test whether SRM assays are sufficiently quantitative and reliable to probe region-specific neurexin repertoires in a complex sample we surveyed eight anatomically defined brain regions from mice at postnatal day 30 (Figure 2A). Importantly, all 17 neurexin forms targeted by the SRM assays could be reliably detected across the brain regions interrogated. In independent measurements from 4 animals using all PTPs described in Figure 1A, we observed close agreement in the observed relative isoform distributions confirming the reproducibility of the approach (Figure 2A).

**Figure 2—figure supplement 1.**
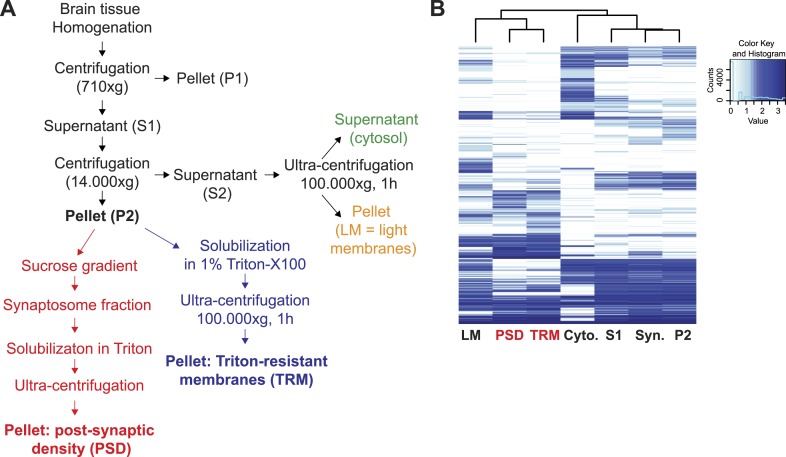
Preparation of synaptic proteins enriched Triton-Resistant-Membranes (TRM) from mouse brain. (**A**) Workflow of the preparation of Triton X-100 resistant membranes (TRM) from brain tissues for enrichment of synaptic proteins. (**B**) Hierarchical clustering of log-transformed protein normalized spectral counts was performed using Ward's algorithm and the Euclidian distance metric. Subsequently, a heat map was created using the gplots R package. To include proteins with zero spectral counts, in one or more conditions, all spectral counts were incremented by one pseudo-count. Heat map diagram and hierarchical clustering of proteins identified in TRM samples and post-synaptic density (PSD) samples prepared using a standard protocol. **DOI:** http://dx.doi.org/10.7554/eLife.07794.012

**Figure 2—figure supplement 2:**
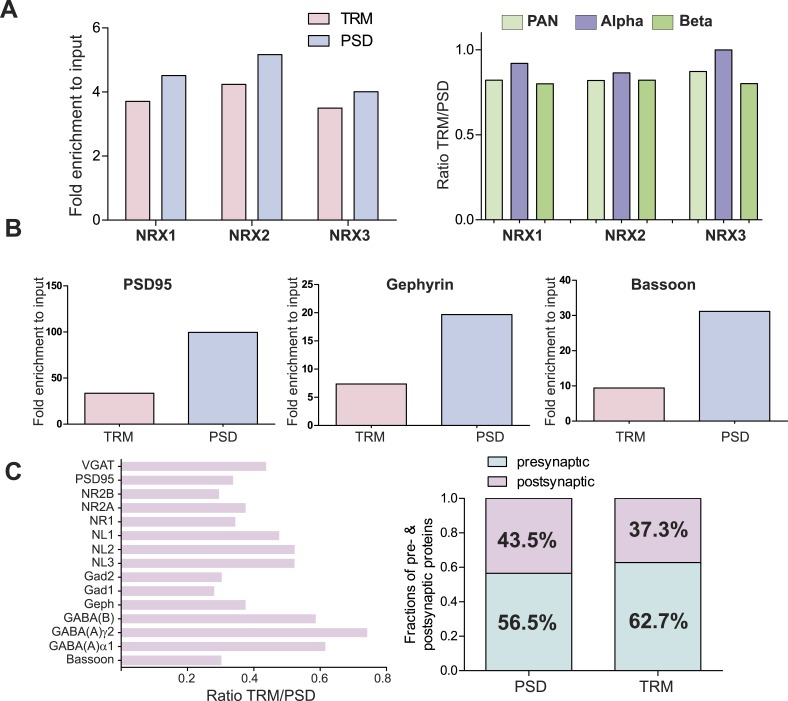
Comparison of enrichments of neurexins and other synaptic proteins in TRM and Postsynaptic-Density (PSD) preparations. (**A**) SRM based quantification of the enrichment of neurexin proteins in TRM and PSD samples (left diagram). **.** In both preparations comparable enrichment (∼4 fold) could be observed. No bias in enrichment of alpha and beta isoforms was observed between two preparations (right diagram). (**B**) Quantitative comparison of the enrichment of core PSD proteins in TRM and purified post-synaptic density (PSD) fractions. Relative enrichments compared to input for PSD95 (Dlg4), Gephyrin, and Bassoon were determined using SRM assays. Purified PSD fraction showed stronger enrichment of core post-synaptic density proteins. (**C**) Ratios of synaptic protein enrichment as determined by SRM assays. For all measured synaptic proteins, higher enrichment could be observed in the purified PSD fraction (right diagram). The enrichment shows significant differences between different synaptic. **DOI:** http://dx.doi.org/10.7554/eLife.07794.013

Interestingly, NRX1, NRX3pan, and NRX3alpha showed only modest protein level fluctuations across the brain regions examined. By contrast, we discovered highly significant differences in expression for NRX2 and NRX3beta variants as well as usage of specific splice insertion in isoforms derived from all three *Nrxn* genes. Region-specific alterations in the proteins derived from primary transcripts show up to threefold increased representation of the NRX2beta and 3beta variants in the cerebellum as compared to other brain regions examined (Figure 2A). Notably, this elevation of beta-variants has only a modest impact on the pan-neurexin level in the cerebellum. This suggests that beta-variants make only a small contribution to the total neurexin pool (see below for absolute quantification of alpha and beta isoform levels).

Insertions at NRX1 AS3 showed little variation across brain regions, whereas other splice insertions exhibited highly differential expression (Figure 2B). Interestingly, the recently identified AS6 insertion in NRX3alpha (Treutlein et al., 2014; Schreiner et al., 2014b) is detected in a rostro-caudal gradient across brain regions. Also of note, there is a significant elevation of AS4 insertion-containing NRX1 and NRX2 proteins in the cerebellum, a site that expresses particularly high levels of an AS4-specific ligand (Uemura et al., 2010).

Hierarchical clustering analysis of the splice variant expression across the mouse brain revealed two major groups with regional co-regulation: one contains variants with AS4 insertion, the other containing variants with AS3 insertion. Remarkably, AS6 containing variants of NRX1 and NRX3 do not segregate into one cluster as this is the case for AS3 and AS4 insertions, indicating independent regulation of AS6 insertion in the *Nrxn*1 and *Nrxn*3 genes (Figure 2C).

### Absolute quantification of neurexin variants

**Figure 3.**
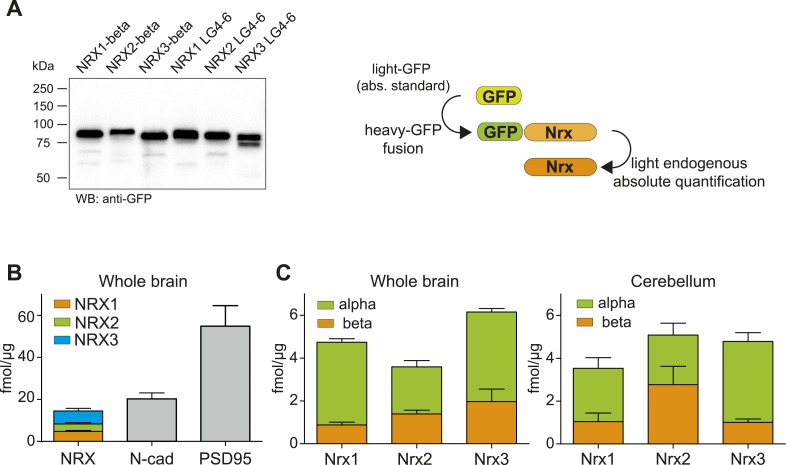
Absolute quantification of endogenous neurexin isoforms. (**A**) Western blot for recombinant proteins and SRM-based quantification approach using in vitro translated heavy amino acids labeled standards fused to GFP and as heavy standards in SRM assays. For absolute quantification of NRX-beta isoforms full-length NRX-beta fused to GFP was used as heavy standard. For absolute quantification of NRX-alpha proteins, the neurexin-repeat 3 containing laminin-G-like domains 4 and 6 (LG4-6) fused to GFP was used as heavy standards. (**B**) Absolute amounts of NRX1,2,3, N-cadherin, and PSD95 in synaptic protein enriched fractions from whole brain of P30 mice. Pan NRX1,2,3 levels were determined using pan NRX peptides derived from in vitro translated NRX-alpha and beta standards. Shown are means ± SD. NRXs were measured with two independent heavy standards (alpha and beta), in two technical replicates, from 2 animals, n = 2. N-cad and PSD95 were measured in two technical replicates, from 2 animals, n = 2. (**C**) Absolute quantification of NRX-alpha and beta in synaptic protein enriched samples from whole brain and cerebellum of adult (P30) mice (n = 2 mice). Quantification was performed with NRX-alpha and beta heavy standards spiked into the brain samples. The amount of NRX-beta in samples measured with alpha-constructs was calculated by subtraction of measured NRX-alpha amount from measured NRX-pan amount. In addition, the amount of NRX-alpha was calculated in samples measured with beta heavy standards. Means ± SD of these independent determinations are shown. **DOI:** http://dx.doi.org/10.7554/eLife.07794.014

To understand which protein isoforms derived from the three *Nrxn* genes are most abundant and to dissect stoichiometry of neurexin proteins vis-à-vis other synaptic components, we performed absolute quantification of NRX proteins with isotope-labeled standards. Since approaches based on reference peptides are prone to artifacts resulting from digestion variations (Carr et al., 2014) we spiked intact heavy protein standards into samples before digestion (Simicevic et al., 2013). The standards were in vitro translated as isotope-labeled GFP-fusion proteins (Figure 3A). Together with an accurately quantified amount of an unlabeled GFP protein standard, the heavy protein standards were combined with the brain extracts of interest, proteolytically digested, and PTPs quantified in separate assays by SRM (an approach that has been referred to as Protein Standard Absolute Quantification ‘PSAQ’ (Brun et al., 2007). Absolute amounts of the heavy, in vitro-produced GFP fusion proteins were determined via peptides derived from the light GFP protein standard. The heavy standard in turn served to deduce absolute amounts of the endogenous proteins in the brain extracts using target protein-specific peptides. To assess the sensitivity of these assays, limits of detection were determined for each peptide used for absolute quantification (Figure 3—figure supplement 1 and Supplementary file 1B). To minimize the contribution of any unlabeled protein contaminants present in the in vitro translated protein standards, the ratios of standard-derived to endogenous peptides for each SRM-assay were titrated to 0.5 and 2 (see ‘Materials and methods’ for details).

**Figure 3—figure supplement 1.**
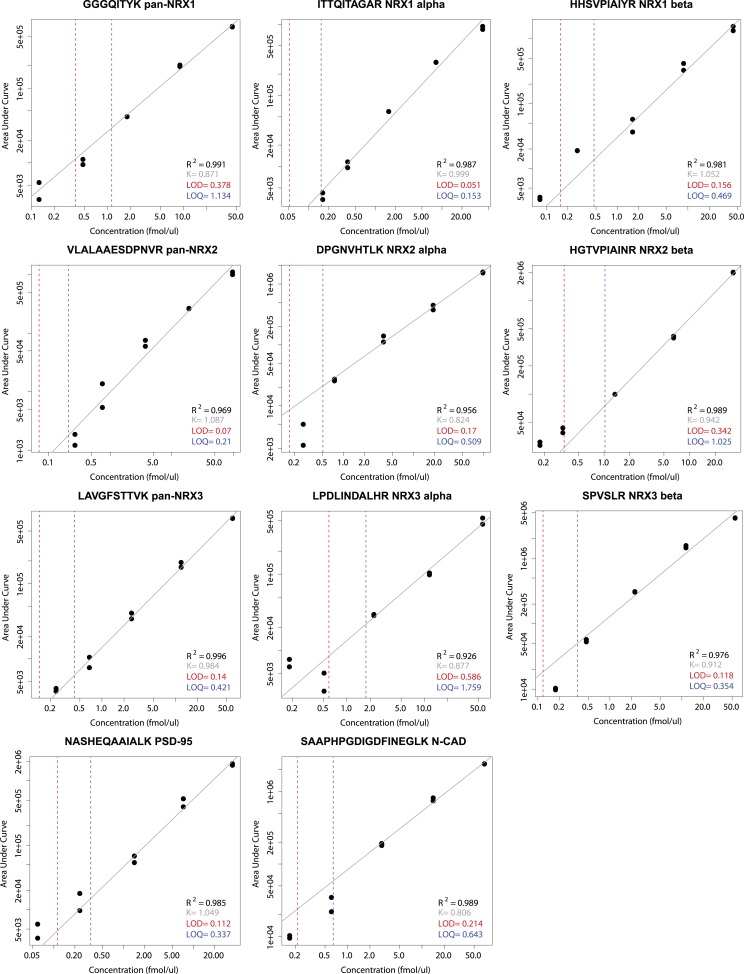
Examples for the determination of lower limits of detection (LOD) and quantification (LOQ) for selected NRX1-3, PSD95 and N-CAD peptides used in this study for absolute quantification. Peptide-specific standard curves were derived by spiking each digested synaptic protein-construct at a concentration range of five values into a digested mixture of mouse whole brain TRM protein extract complemented with GFP protein standard. The measurements were performed in two biological and two technical replicates, including 3 technical replicates of the whole brain protein extract and GFP protein standard mixture alone, here used to assess the background noise level. Lower limits of quantification and lower limits of detection were then established according to the blank and low concentration sample method. **DOI:** http://dx.doi.org/10.7554/eLife.07794.015

Based on these experiments, we found that the amount of total NRX proteins in synapse-enriched fractions from whole brain was approximately 18 fmol/μg protein (Figure 3B). This was similar to the amounts of N-cadherin (∼20 fmol/μg) and approximately threefold lower than amounts of PSD95 (54 fmol/μg), one of the most abundant proteins in the postsynaptic density of glutamatergic synapses. For all three neurexins, the alpha-isoforms represented the major protein variants, whereas beta variants were 2- to 4-fold less abundant (NRX1beta: 0.87 ± 0.36 fmol/μg, NRX1-alpha: 3.86 ± 0.46 fmol/μg, NRX2-beta: 1.39 ± 0.49 fmol/μg, NRX2-alpha: 2.19 ± 0.83, NRX3-beta: 1.97 ± 1.6 fmol/μg, NRX3-alpha: 4.18 ± 0.45 fmol/μg, n = 4, ±SD). Importantly, independent measurements with in vitro translated alpha and beta standard proteins for each primary NRX isoform (each quantified based on different PTPs) yielded highly similar results for pan-NRX amounts as well as the contents of alpha and beta isoforms. This further confirms the accuracy of this method. Based on previous estimates for the number of N-cadherin molecules per synapse (Wilhelm et al., 2014), we estimate the average number of neurexin molecules to be 7–16 for NRX-beta and 18 to 35 for NRX-alpha per synapse.

### Profiling recognition specificity of neurexin receptors

**Figure 4.**
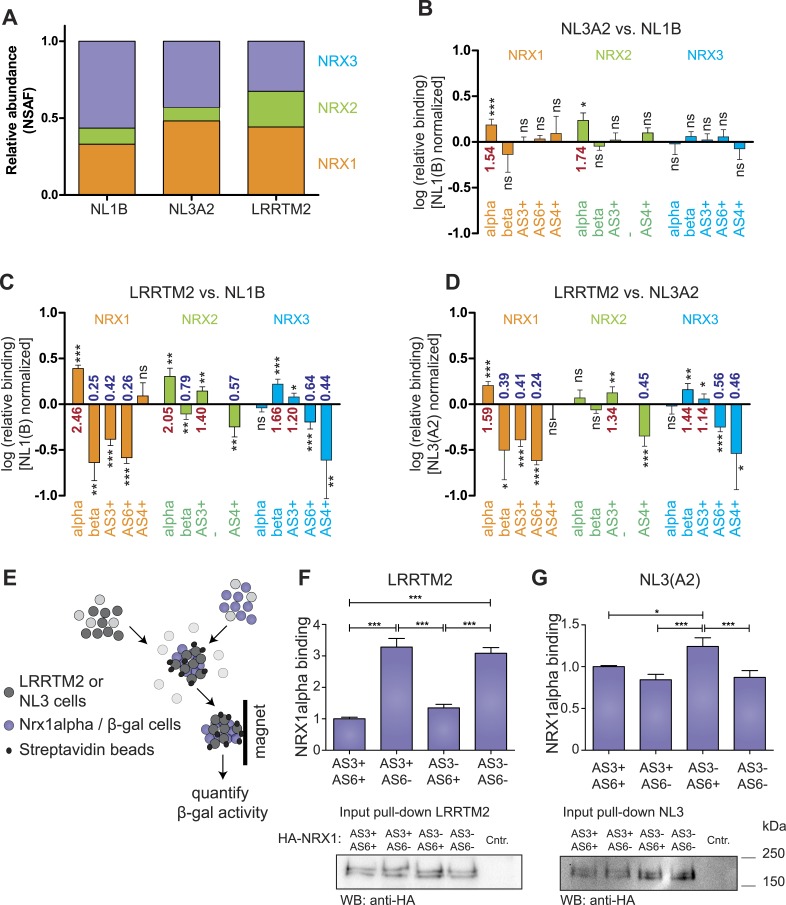
Receptor recognition specificity profiling with splice site-specific SRM assays. **(A)** Ratios of normalized spectral abundance factor (NSAF) of NRX1, 2, 3-specific peptides recovered from NL1B, NL3A2, LRRTM2-affinity matrices detected in shotgun experiments. Means of the measurements from three independent experiments are shown. **(B–D)** Pairwise comparison of neurexin protein variant binding to immobilized ligands determined by SRM. Relative peptide recovery normalized to pan-peptides (for alpha-, beta-, and AS4) or alpha-peptides (for AS3 and AS6) is shown as log10 of the ratio of peptide amounts recovered (NL3A2/NL1B; LRRTM2/NL1B; LRRTM2/NL3A2). n = 6 (measured in duplicates), ±SD. Analysis of significance was performed using nonparametric one-way ANOVA test with Bonferroni's Multiple Comparison Test (*p ≤ 0.05, **p ≤ 0.01, ***p ≤ 0.001). **(E–G)** Quantitative adhesion assays for NRX1alpha splice variant-expressing cells and LRRTM2 or NL3A2-expressing cells. LRRTM2 and NL3A2 cells co-express a surface biotin tag for isolation using streptavidin-magnetic beads. NRX1-alpha variants co-express beta-Galactosidase for quantification. (n = 4 independent experiments, each with 3 replicates measured in triplicates), ±SD. Pairwise comparison was performed using nonparametric one-way ANOVA test with Bonferroni's Multiple Comparison Test (*p ≤ 0.05, **p ≤ 0.01, ***p ≤ 0.001). The expression level of NRX1-alpha constructs was probed by Western blotting. **DOI:** http://dx.doi.org/10.7554/eLife.07794.016

The ability to detect and accurately quantify individual neurexin isoforms opens the possibility to systematically explore the binding selectivity of endogenous neurexins with synaptic receptors. Thus, we profiled recognition specificity of neurexin isoforms for three different postsynaptic receptors detected at glutamatergic synapses: neuroligin-1 (NL1B, containing splice insertion B), neuroligin-3 (NL3A2, containing splice insertion A2), and the leucine-rich transmembrane protein 2 (LRRTM2) (Chih et al., 2006; Budreck and Scheiffele, 2007; de Wit et al., 2009; Siddiqui et al., 2010). Affinity matrixes containing recombinant postsynaptic receptor proteins were incubated with protein extracts from mouse brain and bound proteins were analyzed by mass spectrometry. Based on the normalized spectral abundance factor (NSAF) (Zybailov et al., 2006) of proteins identified by shotgun analysis, neurexins were amongst the most abundant proteins associated with the receptor proteins. Proteins derived from all three *Nrxn* genes were recovered on each of the binding partners. Interestingly, LRRTM2 beads led to a more robust isolation of NRX2-derived peptides as compared to NL1B and NL3A2 (Figure 4A). Quantification by spectral counts provides a good estimate of total protein isoforms derived from the individual *Nrxn* genes but enables only limited detection of isoforms identified by short unique sequence elements, such as beta isoforms or specific splice variants (e.g., average spectral counts for NRX-beta isoforms were ≤3 in three independent pull-down experiments). By contrast, SRM analysis revealed highly significant differential recovery of specific neurexin isoforms on the postsynaptic ligands. Comparison of neurexin isoforms associated with NL1B and NL3A2 matrices revealed preferential binding of NRX1 and 2 alpha forms to NL3A2 over NL1B. The selectivity of interactions was even more significant when comparing binding to LRRTM2 vs NL1B or NL3A2. LRRTM2 showed higher recovery of NRX1alpha and 2 alpha isoforms and lower recovery of NRX1beta and 2beta isoforms. Most importantly, NRX1alpha variants containing AS3 and AS6 insertions were significantly de-enriched in LRRTM2 pull-downs as compared to NL1B and NL3A2. This suggests a major role for neurexin alternative splicing in differential association with these postsynaptic ligands.

To directly test the impact of AS3 and AS6 splice insertions on NRX1alpha—LRRTM2 interactions we applied a quantitative adhesion assay in heterologous cells (Schreiner and Weiner, 2010). We compared adhesive interactions between cells expressing NRX1alpha variants containing different combinations of alternative insertions at AS3 and AS6 and cells expressing LRRTM2 or NL3A2 (Figure 4F). These experiments demonstrated a threefold increase in adhesive interactions of NRX1alpha isoforms lacking AS6 insertions as compared to the AS6+ isoforms. Presence or absence of the insertion at AS3 did not affect adhesion to LRRTM2 cells in this assay. By contrast, for NL3A2 presence of the AS6+ insertion in NRX1alpha resulted in slightly increased adhesion and isoforms containing AS6 but lacking AS3 showed the strongest adhesive interactions. These experiments uncover a significant differential regulation of neurexin interactions with two different receptors through a splice code at two alternatively spliced segments that had not previously been implicated in molecular recognition events.

## Discussion

We developed an approach for the relative and absolute quantification of protein isoforms derived from highly diversified gene families in complex samples. Recent work uncovered a key function for alternative splicing in the regulation of neuronal recognition events. Remarkably, even small modifications in the amino acid sequence translate into switch-like alterations in cell surface recognition events (Chih et al., 2006; Uemura et al., 2010). Thus, the development of tools that enable a quantitative assessment of closely related protein isoforms in complex samples is of central importance.

We validated an array of isoform-specific SRM assays for quantitative detection of neurexin variants resulting from alternative splicing at segments AS3, 4, and 6. The same approach can be extended to the remaining sites modified by alternative splicing (AS1, 2, 5). While we focused in this study on tryptic peptides the use of additional proteases will expand the repertoire of peptides available for analysis, making the quantitative detection of protein isoforms in complex samples even more reliable (Guo et al., 2014; Meyer et al., 2014). The detection of specific splice isoforms at the protein level is particularly important for the recently discovered AS6 variants which are rare (Schreiner et al., 2014b), and there has been no evidence that such isoforms exist on the protein level and have any functional relevance.

In the pull-down experiments with three known ligands we profiled their selectivity for particular endogenous NRX isoforms and uncovered a role for insertions at alternatively spliced segments 3 and 6 (AS3 and AS6) in gating interactions with LRRTM2 vs neuroligin-3. Notably, crystallographic studies provide a potential structural basis for this regulation (Chen et al., 2011; Miller et al., 2011). The six amino acids incorporated in AS6+ variants are positioned at a hinge region in the L-shaped NRX1alpha protein (Schreiner et al., 2014b). Thus, the regulation of LRRTM2 binding to laminin-G domain 6 might involve a modification of the flexibility positioning laminin-G domains 1-5 vs the EGF domain 3 and laminin-G domain 6 in the NRX1alpha structure. Moreover, modeling studies predict that NRX1alpha AS3 is positioned in close proximity to an interaction surface with neuroligins (Chen et al., 2011; Tanaka et al., 2012). This might underlie the modulation of NRX1alpha-NL3A2 interactions by AS3 insertions observed in our assays.

Based on our absolute quantification experiments, we come to two important conclusions. First, within individual brain regions, neurexin-alpha isoforms are significantly more abundant than beta isoforms. In fact, many previous studies with exogenously expressed neurexins focused on NRX1beta isoforms but our quantitative analysis demonstrates that these forms are the least abundant of all neurexins in vivo. Considering that neurexin-alpha and beta forms differ in their interactions with receptors (Figure 4 and Boucard et al., 2005; Chih et al., 2006; Siddiqui et al., 2010), this has important functional implications. Second, comparison of absolute levels of neurexins with other synaptic proteins demonstrates that neurexins are rather abundant synaptic proteins. For example, the total amount of all neurexin proteins is similar to that of N-cadherin, a highly abundant synaptic protein and about one third of the amount of PSD95, one of the most abundant proteins in glutamatergic synapses.

During the development of our study, we evaluated the use of AQUA synthetic peptides (Gerber et al., 2003) for quantification of brain protein repertoires. We observed that several different synthetic AQUA peptides reporting on the same neurexin isoform often yielded divergent results, making a reliable quantification difficult (data not shown). This is most likely due to the variability in tryptic digests of endogenous proteins (Arsene et al., 2008). To circumvent these problems, we utilized an in vitro recombinant protein expression system, to use surrogate protein-constructs as standards for quantification. This approach is not only more robust and more accurate, but is also cost-effective, can be multiplexed by using multiple different tags in the isotopically labeled function proteins (in addition to GFP) and can be scaled to screen large numbers of different proteins and isoforms. Thus, the experimental pipeline established here is readily transferable to any protein class of interest.

Finally, our SRM approach provides an attractive alternative to isotope labeling-based massspectrometry approaches such as SILAC. Notably, SILAC labeling has been most frequently applied to cell culture or microorganisms. This powerful technique has been extended to higher organisms, by using labeled cell lines as an internal standard for quantitation or raising rodents for multiple generations on a diet with heavy isotope-labeled amino acids (Kruger et al., 2008). While methods are well suited to assess overall proteome modifications it remains challenging to measure proteins of low abundance or protein isoforms in a consistent manner and across large sample numbers with reasonable throughput, due to fact that neuronal tissues are complex and heterogeneous. In most instances, the SRM-assays developed and validated here for mouse tissues can be directly transferred to assessing protein levels in small amounts of biopsy material from human patients or postmortem brain tissue. This is particularly interesting for the *Nrxn* gene family as *Nrxn*1 mutations are associated with autism and schizophrenia in the human population (Reichelt et al., 2012). Given the complexity and size of the mammalian neurexin genes it is difficult to predict the impact of such mutations on the expression and function of the neurexin proteins. In the future, the approach established here can be readily applied to iPSC-derived human neurons, biopsy or postmortem tissue and enables exploration of the impact of such mutations on the neurexin isoform repertoires.

## Materials and methods

### Sample preparation

Brain regions were dissected, weighed, and homogenized in a glass-Teflon homogenizer in 1:20 wt/vol of solution A (0.32M sucrose 1 mM MgCl2 0.5 mM CaCl2 1 mM NaHCO3 complemented with Roche ‘Complete’ protease inhibitors). Homogenates were centrifuged at 1,400×*g* at 4°C for 10 min. Supernatants were re-centrifuged (14,000×*g* at 4°C for 10 min) and pellets re-suspended in Triton X-100 buffer (12 mM Tris–HCl, pH8.1, 1% Triton X-100 complemented with protease inhibitors), rotated at 4°C for 10 min on a rotator. Samples were then centrifuged at 100,000×*g* at 4°C for 1 hr. The pellets representing TRM were dissolved in 50 mM ammonium bicarbonate, 1% sodium deoxycholate (DOX). Protein concentrations were determined using the BCA assay (Pierce).

### DNA constructs and in vitro-expression of heavy protein standards

cDNAs encoding the polypeptides of interest were fused in frame with a cDNA encoding GFP in a modified SP6-pF3A WG(BYDV)-Flexi vector (Promega). Mouse cDNA constructs encoding the following proteins were used (always lacking the N-terminal signal sequence): NRX1-beta AS4+ (containing the alternative insertion at AS4), NRX2-beta AS4+, NRX3-beta AS4-, the third neurexin-repeat (containing laminin-G-domain 5, EGF-like domain 3 and laminin-G domain 6) of NRX1, 2 and 3-alpha, each containing insertions at AS4 and AS6, full-length mouse PSD95, N-cadherin lacking the pro-domain.

Isotopically labeled proteins were expressed using the TnT-SP6 Wheat Germ High Yield Master Mix Minus Amino Acids (Promega). An 18 amino acid mix (80 µM each final concentration) was combined with (^13^C, ^15^N)-isotopically labeled Arginine and Lysine (Cambridge Isotope Laboratories) to a 1 mM each final concentration and processed according to the manufacturer's instructions. Validation of *in vitro*-expressed heavy-labeled proteins was performed by Western blot using mouse anti-GFP primary antibodies (Santa Cruz Biotechnology).

For proteolytic digest, the in vitro translated proteins were reduced in 5 mM Tris-(2-carboxyethyl)-phosphine hydrochloride (TCEP) at 95°C for 10 min, alkylated in 10 mM iodoacetamide at 25°C for 30 min in the dark, and incubated with 12.5 mM N-acetyl-cysteine at RT for 10 min. Samples were then incubated with 12.0 μg/ml trypsin (V511C-Promega) overnight at 37°C. The resulting tryptic peptides were purified by C18 using Macro Spin columns (The Nest Group) and re-suspended in 2% acetonitrile 0.15% formic acid.

### In vitro-expressed synaptic protein identification

Prior to mass spectrometric analyses, digested peptides (1.5 µg) were separated by an EASY nano-LC system (Proxeon Biosystems, Thermo-Scientific) equipped with a reverse phase HPLC column (75 µm × 30 cm) packed in house with C18 resin (Reprosil-AQ Pur, 1.9 μm, Dr Maisch) using a linear gradient from 95% solvent A (98% water, 2% ACN, 0.15% formic acid) and 5% solvent B (98% ACN, 2% water, 0.15% formic acid) to 30% solvent B over 90 min at a flow rate of 0.2 μL/min. For LC-MS/MS analysis of separated peptides a LTQ-Orbitrap Velos and Elite mass spectrometer equipped with a nanoelectrospray ion source (both Thermo Fisher Scientific) and a custom made column heater set to 60°C. Each MS1 scan was followed by collision-induced-dissociation of the 20 most abundant precursor ions with dynamic exclusion for 60 s. Total cycle time was approximately 2 s. For MS1, 10E6 ions were accumulated in the Orbitrap cell over a maximum time of 300 ms and scanned at a resolution of 30,000 (120,000 for Elite) FWHM (at 400 m/z). MS2 scans were acquired at a target setting of 10,000 ions, accumulation time of 25 ms. The mass selection window was set to 2 Da and singly charged ions and ions with unassigned charge state were excluded from triggering MS2 events. Besides, the normalized collision energy was set to 35% and one microscan was acquired for each spectrum.

The acquired raw-files were converted to the mascot generic file (mgf) format using the msconvert tool (part of ProteoWizard, version 3.0.4624 (2013-6-3)). Using the MASCOT algorithm (Matrix Science, Version 2.4.0), the mgf files were searched against a concatenated target-decoy protein sequence database, comprised of target sequences and decoy entries as follows: (a) target sequences/entries: from mouse (Mus musculus—SwissProt, www.uniprot.org, release date 12/05/2012, canonical and isoform sequences), all NRX alternatively spliced segments generated in silico based on a PacBio sequencing data splicing model (Schreiner et al., 2014b); (b) a set of common contaminant protein sequences as defined in the MaxQuant software (Cox and Mann, 2008); (c) decoy entries: the reversed target entries were generated using the SequenceReverser tool from the MaxQuant software (Version 1.0.13.13). The final database contained 33,364 entries in total. The MASCOT search criteria were set as follows: 10 ppm precursor ion mass tolerance, 0.6 Da fragment ion mass tolerance, full tryptic specificity was required (cleavage after lysine or arginine residues unless followed by proline), up to 2 missed cleavages were allowed, carbamidomethylation (C), were set as fixed modification and oxidation (M) as a variable modification. Next, the database search results were imported to the Scaffold software (version 4.3.2, Proteome Software Inc., Portland, OR) and the protein false identification rate was set to 1% based on the number of decoy hits. Specifically, peptide identifications were accepted if they could achieve an FDR less than 1.0% by the scaffold local FDR algorithm. Protein identifications were accepted if they could achieve an FDR less than 1.0% and contained at least 1 identified peptide. Proteins that contained similar peptides and could not be differentiated based on MS/MS analysis alone were grouped to satisfy the principles of parsimony. Proteins sharing significant peptide evidence were grouped into clusters.

### Selected reaction monitoring assays

All samples were analyzed on a TSQ-Vantage triple-quadrupole mass spectrometer coupled to an Easy-nLC (Thermo Fisher, Scientific) equipped with a heated (60°C) reverse phase HPLC column (75 µm × 30 cm) packed in house with C18 resin (Reprosil-AQ Pur, 3 μm, Dr Maisch). In each injection an equivalent of 1.5 μg of synaptic protein extract, including purified synaptic heavy-labeled purified protein fusion constructs and the GFP standard were separated using a linear gradient from 95% solvent A (98% water, 2% ACN, 0.15% formic acid) and 5% solvent B (98% ACN, 2% water, 0.15% formic acid) to 30% solvent B over 90 min at a flow rate of 0.2 μl/min. The mass spectrometer was operated in the positive ion mode using ESI with a capillary temperature of 275°C, a spray voltage of +2200 V. All the measurements were performed in an unscheduled mode and a cycle time of 5 s. A 0.7 Dalton mass selection window was set for parent- (Q1) and product- (Q3) ion isolation. Fragmentation of parent-ions was performed in Q2 at 1.2 mTorr. Each SRM assay was optimized regarding collision energies, parent ion masses and fragment ion selection in pilot experiments using pure heavy peptide reference samples and the Skyline software (v.2.4) (Carr et al., 2014). Generally, singly charged peptide fragment ions of the y-ion series with a mass higher than the precursor ion mass to charge ratio were preferably monitored, unless otherwise stated. A complete list of all monitored transitions is provided in Supplementary file 1A. For relative quantification, 5 μg of TRM proteins were digested and a mix of synthetic heavy-labeled peptides (JPT Peptide Technologies GmbH, Berlin, Germany) at the final concentration of 50 fmol/µl was spiked in the samples prior to injection. For absolute quantification, 5 μg of TRM proteins were supplemented with an equivalent of 0.05 μl of in vitro-expressed heavy-labeled protein construct mixture (which includes the wheat germ lysate) along with the GFP protein standard (Ray Biotechnologies) at the final concentration of 100–200 fmol/µl, prior to resuming the digestion protocol described above.

### Data analysis and absolute quantification of synaptic proteins

Calculation of absolute levels of synaptic proteins was performed using a two-step procedure as outlined in Figure 3A. All data analyses were carried out using the Skyline software (v. 2.4). Peptide identification and peak-area integration of the GFP tag peptides and targeted synaptic peptides as well as their transitions were manually verified in Skyline. Individual standard curves were established for all synaptic protein peptides using a concentration range of five values recorded in two biological and two technical replicates (Figure 3—figure supplement 1). Lower limits of quantification and lower limits of detection were established using the blank and low concentration sample method (Addona et al., 2009).

### Label-free quantitative experiments of the shotgun data

For label-free quantification, the generated raw files were imported into the Progenesis LC-MS software (Nonlinear Dynamics, Version 4.0) and analyzed using the default parameter settings. MS/MS-data were exported directly from Progenesis in mgf format and analyzed using Mascot, searching the same target-decoy databases as specified above. The search criteria were set as follows: 10 ppm precursor ion mass tolerance, 0.6 Da fragment ion mass tolerance, full tryptic specificity required (cleavage after lysine or arginine residues); maximum 2 missed cleavages; fixed modification: carbamidomethylation (C), variable modification: oxidation (M). Results from the database search were imported into Progenesis. The database search results were filtered, limiting the peptide and protein level FDR to 1%. The Progenesis analysis results were further processed using the SafeQuant R package to obtain protein relative abundances. This analysis included, global data normalization by equalizing the total MS1 peak areas across all channels, summation of MS1 peak areas per protein and LC-MS/MS run, followed by calculation of protein abundance ratios and testing for differential abundance using empirical Bayes method.

### Receptor recognition specificity profiling

For the recognition specificity profiling experiments, ectodomains of mouse neuroligin-1 containing insertion B (NL1B), mouse neuroligin-3 containing insertion A2 (NL3A2), and mouse leucine-rich repeat transmembrane protein 2 (LRRTM2) were expressed as SNAP-tagged proteins using the pDisplay expression vector. For expression of each construct, four 15-cm plates of HEK293T cells were transfected, conditioned media were collected 48 hr post-transfection, and incubated with 100 μl of SNAP-capture agarose resin (NEB) overnight. This results in covalent binding of the ‘bait’ proteins to the agarose beads. Beads were washed with pull-down buffer (50 mM Tris–HCl, pH7.5, 150 mM NaCl, 10% glycerol). For pull-down, one brain of an adult (P25-P30) mouse (ca. 0.4g) was homogenized in 20 ml using a glass-homogenizer in pull-down buffer completed with protease inhibitors (Roche), 1% Triton X-100, and 2 mM CaCl_2_. Homogenate was centrifuged at 20,000×*g* for 30 min at 4°C. 5 ml of the supernatant was added to each ‘bait’ protein-bead preparation, and incubated overnight. On the next day, beads were washed 4 times with pull-down buffer supplemented with 0.1% Triton X-100, and 2 mM CaCl_2_, and bound proteins were eluted from the beads with 50 mM ammonium bicarbonate, 1% sodium deoxycholate and prepared for mass spectrometry as described above.

### Adhesion assays

Adhesion assays were performed essentially as described (Schreiner and Weiner, 2010) with some modifications to the protocol. 1 × 106 K562 cells were nucleofected (Amaxa) with 5 μg of ‘prey’ and ‘bait’ DNA mixes. ‘Prey’ mixes: NRX1-alpha (with different combination of insertion at AS3 and AS6) or control plasmid 3 μg, beta-Gal 1.5 μg, RFP 0.5 μg (transfection control). ‘Bait’ mixes: LRRTM2, NL1(B), or NL3(A2) 3 μg plus AP-GFP 1.5 μg and BirA-ER 0.5 μg. After nucleofection, cells were grown in 2.5 ml DMEM in 6 well plates (one nucleofection per well). 24 hr post-nucleofection, 10 μM biotin was added to cells transfected with ‘bait’ mixes. On the next day ‘bait’ cells (5 wells) were pooled, washed 1x with DMEM (without serum), and re-suspend in 10 ml DMEM (without serum) in 15-ml tube. 75 μl neutravidin-magnetic beads (Pierce) were added and incubated for 15 min (overhead shaker). During pre-incubation of ‘bait’ cells with magnetic beads ‘prey’ cells (one well for each NRX1-alpha construct or control) were collected in 15-ml tubes, spun down, and re-suspended in 1.5 ml DMEM (without serum). To each tube with ‘prey’ cell suspension, 2 ml of ‘bait’ cells/magnetic beads suspension was added. Suspension mixes were aliquoted (1 ml) in round bottom 2 ml tubes. One 200 μl aliquot was taken (input). Suspensions were incubated for 60 min at RT on an overhead shaker. Magnetic beads were washed once with 1 ml DMEM and bound cells were lysed in 100 μl of 1X passive lysis buffer (Promega) complemented with protease inhibitors. Input samples (200 μl) were lysed by adding 100 μl of 3x passive lysis buffer.

Beta-gal activity was measured in triplicates: 100 μl of beta-Gal buffer (100 mM phosphate buffer, pH7.4, 2 mM MgCl_2_, 50 mM beta-mercaptoethanol, 1 mg/ml ONPG) were pre-pipetted in a 96 well plate and 20 μl of lysates were added per well. Plates were incubated at 37°C. Absorbance was measured at 415 nm and relative binding was calculated from background-subtracted values.

## Additional files

Supplementary file 1. Lists of transitions and limits of detection and quantification for proteotypic peptides used in this study. (**A**) Complete list of monitored transitions of peptides for neurexin and other proteins used in this study. (**B**) Complete list of Limits of detection (LOD) and quantification (LOQ) for peptides derived from in vitro expressed neurexin constructs used for absolute quantification of neurexin isoforms in this study. **DOI:** http://dx.doi.org/10.7554/eLife.07794.017
