# Supplementary material for: Quantitative isoform-profiling of highly diversified recognition molecules
Source: eLife. 2015 May 18;4:e07794. doi: 10.7554/eLife.07794 (PMC4489214; doi:10.7554/eLife.07794)
Supplement: Related texts

# Article commentary: Decision letter

**DOI:** 10.7554/eLife.07794.018

**Affiliations:** Stanford School of Medicine, United States

eLife posts the editorial decision letter and author response on a selection of the published articles (subject to the approval of the authors). An edited version of the letter sent to the authors after peer review is shown, indicating the substantive concerns or comments; minor concerns are not usually shown. Reviewers have the opportunity to discuss the decision before the letter is sent (see review process). Similarly, the author response typically shows only responses to the major concerns raised by the reviewers.

Thank you for sending your work entitled “Quantitative Isoform-Profiling of Highly Diversified Recognition Molecules” for consideration at *eLife*. Your Tools and Resources article has been favorably evaluated by Aviv Regev (Senior editor), a Reviewing editor, and three peer reviewers.

All three reviewers are highly enthusiastic about publication in *eLife*.

Reviewer 1 said that: “This is a very nice, clear, and crisp paper with a few very interesting insights on a very interesting protein family. The SRM workflow established by the authors looks powerful and will be interesting to a wide spectrum of potential users. The key data on the relative and absolute abundance of different Nrxns and their different association with ligands are very interesting and will also be received with substantial interest.”

Reviewer 2 stated: “While technical in scope, this work provides novel insights into receptor isoform expression and splicing-dependent recognition for synaptic ligands. The proteomics components of this work are very strong and represent a significant amount of work. Especially the absolute quantification experiments using accurately quantified GFP and GFP fusions generated in vitro was quite clever and could be easily extended to similar biological questions. Obtained results are systematically analyzed and initial validations performed that will guide future in-depth mechanistic investigations. Overall, I felt this work presented a substantial amount of work and a benchmark of how to use target proteomics strategies to obtain novel insights into isoform-specific biology. I am quite positive to support publication of this work in *eLife*.”

Reviewer 3: “In this manuscript titled ‘Quantitative Isoform-Profiling of Highly Diversified Recognition Molecules’, Schreiner and colleagues report a mass spectroscopy based method that permits quantitative measurement of different splice variants and isoforms of the highly diverse neurexin cell adhesion protein family. They do so by developing a workflow for targeted mass spectroscopy that is based on selected reaction monitoring (SRM) signatures of individual variants. Their results provide quantitative measurement of neurexins from different regions of mouse brain and reveal the relative abundance of certain isoforms. In general the experiments are well executed and the strategy is elegant. The possibility of extending this type of analyses to other protein families is very attractive.”

However, we request the following textual and figure revisions before acceptance.

1) The Introduction is not accessible to a broad audience. It is full of jargon and does not provide the important background on the targeted mass spectroscopy. Having an informative and general Introduction is critical for the readers to understand the technical aspects of this method. Instead of the sections that review other approaches on mass spectroscopy in extensive detail, the authors should explain with better clarity the reasoning behind developing the SRM approach.

2) The authors have prepared TX-100 insoluble membrane fractions for their analysis. They have a supplemental figure showing the workflow, however no data is provided to display what this fractionation step selects for. Are NRXs and other synaptic proteins enriched in these membranes? Are these presynaptic and/or postsynaptic fractions? Do these fractions include all types of synapses (excitatory and inhibitory) or this method only enriches for one type over the other? These are important distinctions to make otherwise the data that is provided may not reflect the endogenous levels of NRX variants in the brain, but just in these detergent insoluble fractions.

3) NRXs are thought to be primarily presynaptic. So the NRX species in a brain region may be originating from the innervations from another region. This is not mentioned in the paper and is an important point to make.

4) It was not completely clear how many of the >1000 isoforms (see Introduction) are actually amendable to SRM development. Do all of these isoforms generate peptides that could be used in SRM-MS, based on common restrictions of usable peptides? Please comment?

5) In the subsection headed “Profiling recognition specificity of Neurexin receptors”, why are the authors using spectral counting in their binding studies since SRM assays have already been developed?

6) It was interesting to see that AQUA peptides resulted in poor quantitative data, while the in vitro recombinant protein expression system was able to overcome these issues. Could the authors comment if any of the neurexin receptors are PTM modified in vivo and how this could affect quantification in their assay?

7) In the subsection entitled “In vitro-expressed synaptic protein identification”, it is not completely clear how the database for the mascot searched was generated. Was this a combination of SwissProtein plus NRX sequences based on the authors RNA-seq data?

8) Figure 2A/B: The relative quantification compared to the OB samples was based on all peptides shown in Figure 1?

9) Figure 4B–D: Why are there no error bars on these figures?

10) Figure 3–figure supplement 1: were the two technical replicates averaged? We only see two data points for each concentration in these response curves.

11) Introduction: Asides from random sampling, protein inference is likely an even bigger issue, especially for large protein families with high sequence homology.

12) This sentence is unclear: “AS6 containing variants of NRX1 and NRX3 segregate into different clusters, indicating independent regulation of these isoforms”.

13) In the sentence “for absolute quantification 5 μg of TRM proteins were supplemented with an equivalent of 0.05 μl of in vitro-expressed heavy-labeled protein construct mixture (which includes the wheat germ lysate) along with the GFP protein standard (Ray Biotechnologies) at the final concentration of 100-20 fmol/μl, prior to resuming the digestion protocol described above”, 100-20 fmol/μl: is this 100-200 or 20-100?

# Reply: Author response

**DOI:** 10.7554/eLife.07794.019

*1) The Introduction is not accessible to a broad audience. It is full of jargon and does not provide the important background on the targeted mass spectroscopy. Having an informative and general Introduction is critical for the readers to understand the technical aspects of this method. Instead of the sections that review other approaches on mass spectroscopy in extensive detail, the authors should explain with better clarity the reasoning behind developing the SRM approach*.

We appreciate this concern. We have now significantly reworked this section. We eliminated the general description of other mass-spec approaches and provide a more focused introduction to targeted proteomics.

*2) The authors have prepared TX-100 insoluble membrane fractions for their analysis. They have a supplemental figure showing the workflow, however no data is provided to display what this fractionation step selects for. Are NRXs and other synaptic proteins enriched in these membranes? Are these presynaptic and/or postsynaptic fractions? Do these fractions include all types of synapses (excitatory and inhibitory) or this method only enriches for one type over the other? These are important distinctions to make otherwise the data that is provided may not reflect the endogenous levels of NRX variants in the brain, but just in these detergent insoluble fractions*.

This data was actually included in the original submission. We apologize that it may not have been highlighted appropriately and may have been missed in the review.

To validate the use of Triton-resistant membranes in this analysis all fractions were analyzed by shotgun proteomics and the SRM approach. The data from this analysis is included in Figure 2–figure supplement 1 and Figure 2–figure supplement 2. Using hierarchical clustering analysis of protein abundance determined based on spectral counts we find that at the global level enrichment/ de-enrichment is very similar in the “conventional” postsynaptic density fraction and the Triton X-100 resistant membranes (see Figure 2–figure supplement 1B).

We further examined enrichment of neurexin isoforms, excitatory and inhibitory markers, as well as pre- and postsynaptic proteins to explore whether the method may enrich one specific fraction over another. These comparisons are provided in Figure 2–figure supplement 2.

In brief, we find that enrichment of neurexin 1,2,3 alpha and beta forms is very similar in “conventional” postsynaptic density (PSD) fraction and the Triton X-100 resistant membranes (Figure 2–figure supplement 2A).

We find that the enrichment of pre- and postsynaptic markers is somewhat reduced in the Triton X-100 resistant membranes as compared to PSD preparations but there is no differential enrichment with respect to pre- vs postsynaptic markers (Figure 2–figure supplement 2B).

Finally, by surveying a panel of inhibitory (mostly GABAergic) and excitatory (glutamatergic) markers we do not observe differential enrichment for synaptic proteins associated with synapses of either neurotransmitter phenotype (Figure 2–figure supplement 2C).

This data is summarized in the subsection “Generation and characterization of peptide library and sample preparation” of the revised manuscript.

*3) NRXs are thought to be primarily presynaptic. So the NRX species in a brain region may be originating from the innervations from another region. This is not mentioned in the paper and is an important point to make*.

We thank the reviewers for this remark. We agree that due to the long-distance neuronal projections neurexin expression at mRNA level and the protein localization might differ significantly with respect to brain regions. Since this issue applies to most synaptic proteins we added this important point in the Introduction.

*4) It was not completely clear how many of the >1000 isoforms (see Introduction) are actually amendable to SRM development. Do all of these isoforms generate peptides that could be used in SRM-MS, based on common restrictions of usable peptides? Please comment*?

We now included the coverage of alternatively spliced segments assessed in this study in the Discussion. Moreover, we did highlight the possibility of extending our approach to the analysis of all alternatively spliced segments in neurexins using a combination of proteolytic enzymes (in addition to trypsin which was used in the analysis described in this manuscript).

*5) In the subsection headed “Profiling recognition specificity of Neurexin receptors”*, *why are the authors using spectral counting in their binding studies since SRM assays have already been developed*?

The main reason to also include spectral count data derived from shotgun analysis was to compare the two methods. This comparison highlights the benefits of implementation of targeted-SRM for quantitative mass spectrometric analysis.

*6) It was interesting to see that AQUA peptides resulted in poor quantitative data, while the* in vitro *recombinant protein expression system was able to overcome these issues*. *Could the authors comment if any of the neurexin receptors are PTM modified* in vivo *and how this could affect quantification in their assay*?

We now provide further information on the potential impact of post-translational modifications in the Results section of the manuscript. Please note that the in vitro translated protein standards used for quantification also lack posttranslational modifications (they are generated in a cell-free transcription-translation system). Thus, the poor quantitative data observed for AQUA peptides arises primarily from inefficient digest of endogenous proteins most probably due to the amino acid sequences surrounding the tryptic cleavage sites. Producing the proteotypic peptide from the digest of in vitro expressed protein circumvents this problem to a significant extend.

*7) In the subsection entitled “In vitro-expressed synaptic protein identification”, it is not completely clear how the database for the mascot searched was generated. Was this a combination of SwissProtein plus NRX sequences based on the authors RNA-seq data*?

Yes, we did use a combination of SwissProt and RNA-Seq data. We have now added this information to the Material and methods section.

*8)* *Figure 2*A*/B*: *The relative quantification compared to the OB samples was based on all peptides shown in* *Figure 1*?

Yes. We now stated this in the text (see subsection “Generation and characterization of peptide library and sample preparation”).

*9)* *Figure 4B–D*: *Why are there no error bars on these figures*?

We apologize for this omission. We have now included the appropriate error bars in Figure 4B–D.

*10)* *Figure 3–figure supplement 1**: were the two technical replicates averaged? We only see two data points for each concentration in these response curves*.

The graphs in Figure 3–figure supplement 1 show the dilution curve plot for 1 biological replicate in 2 technical replicates (the two dots per dilution on the graph). For simplicity we did not merge the two biological replicates into one unique figure. However, when calculating the LOQ and LOD values, both biological replicates were taken into account; the resulting values per Neurexin peptide are thus the average of the two biological replicate measurements. We have now clarified this in the figure legend.

*11) Introduction: Asides from random sampling, protein inference is likely an even bigger issue, especially for large protein families with high sequence homology*.

Thanks for this important remark. We now mentioned this problem in the text and cited a paper discussing this issue in detail (Nesvizhskii and Aebersold, “Interpretation of Shotgun Proteomic Data: The Protein Inference Problem”, MCP, 2005).

*12) This sentence is unclear: “AS6 containing variants of NRX1 and NRX3 segregate into different clusters, indicating independent regulation of these isoforms*”.

We reworded this sentence to improve clarity.

*13) In the sentence “for absolute quantification 5 μg of TRM proteins were supplemented with an equivalent of 0.05 μl of in vitro-expressed heavy-labeled protein construct mixture (which includes the wheat germ lysate) along with the GFP protein standard (Ray Biotechnologies) at the final concentration of 100-20 fmol/μl, prior to resuming the digestion protocol described above”*, *100-20 fmol/μl: is this 100-200 or 20-100*?

We thank the reviewers for pointing out this typo. The correct concentration is indeed 100-200 fmol/μl. We corrected this in the text.
